# Seroprevalence of IgM/IgG and Neutralizing Antibodies against SARS-CoV-2 in Unvaccinated Young Adults from Mexico Who Reported Not Having Had a Previous COVID-19 Infection

**DOI:** 10.1155/2024/8871439

**Published:** 2024-02-14

**Authors:** Diana Lourdes Padilla-Bórquez, Mónica Guadalupe Matuz-Flores, Jorge Hernández-Bello, Gabriela Athziri Sánchez-Zuno, Samuel García-Arellano, Edith Oregon-Romero, Melva Guadalupe Herrera-Godina, Guillermo González-Estevez, Norma Patricia Adan-Bante, Jesús Alfredo Rosas-Rodríguez, José Francisco Muñoz-Valle

**Affiliations:** ^1^Instituto de Investigación en Ciencias Biomédicas, Centro Universitario de Ciencias de la Salud (CUCS), Universidad de Guadalajara, Guadalajara 44340, Mexico; ^2^Departamento de Ciencias Químicas, Biológicas y Agropecuarias, Unidad Regional Sur, Universidad de Sonora, Navojoa, Sonora 85880, Mexico

## Abstract

**Background:**

Severe acute respiratory syndrome coronavirus 2 (SARS-CoV-2) is the virus that causes coronavirus disease 2019 (COVID-19). It is estimated that more than half of new infections are transmitted by asymptomatic people; therefore, the isolation of symptomatic people is not enough to control the spread of the disease.

**Methods:**

A total of 171 unvaccinated young adults (18–35 years) from Sonora, Mexico, who underwent a structured survey to identify prior COVID-19 infections, were included in this study. A qualitative determination of anti-SARS-CoV-2 antibodies in serum was performed by lateral flow immunoassay (Certum IgG/IgM Rapid Test™ cassette kit) and neutralizing antibodies were also determined (GenScript cPass assay).

**Results:**

A total of 36 people reported a history of COVID-19 infection, and 135 reported no history of COVID-19. In contrast, 49.6% (67/135) of individuals who had not reported a previous SARS-CoV-2 infection were seropositive to the rapid anti-SARS-CoV-2 antibody test, and 48.1% (65/135) of them had neutralizing antibodies.

**Conclusions:**

These results suggest that in young adults, SARS-CoV-2 infections could be asymptomatic in a high percentage of individuals, which could contribute in part to the slow control of the current pandemic due to the large number of asymptomatic cases that are contagious and that could be a silent spread of the virus.

## 1. Introduction

The severe acute respiratory syndrome coronavirus 2 (SARS-CoV-2) virus identified in late 2019 in Wuhan, China, is the cause of coronavirus disease 2019 (COVID-19) [1, 2]. In February 2020, the International Committee on Taxonomy of Viruses named it SARS-CoV-2, which, in the first instance, was known as the new coronavirus 2019 (2019-nCoV) or human coronavirus 2019 (hCoV-19) [3].

COVID-19 quickly spread worldwide, and by the beginning of March 2020, it was declared a pandemic by the World Health Organization (WHO) [4].

More than 750 million infections and 6.8 million deaths have been attributed to SARS-CoV-2 since the start of the COVID-19 pandemic. Regarding pathogenesis, the spectrum of SARS-CoV-2 infection can range from asymptomatic infection to life-threatening complications of COVID-19 [5]. The most common symptoms are fever, cough, shortness of breath, anosmia, and ageusia which correspond to mild symptoms. Moderate symptoms include clinical or radiographic evidence of lower respiratory tract disease and oxygen saturation ≥94%; severe symptoms include oxygen saturation <94%, respiratory rate ≥30 breaths/min, and lung infiltrates >50%, and critical symptoms include respiratory failure, shock, and multiorgan dysfunction or failure [6–8].

It has been identified that a large part of the population is asymptomatic of the disease; that is, they do not present any apparent clinical symptoms, which can favor the spread of the infection [8]. A high prevalence of asymptomatic infections has been documented in various regions worldwide, encompassing countries across Asia, Europe, Africa [9–12], and the Americas, including Mexico [13]. These individuals, who are primarily mostly young adults [9, 14], workers, health care workers, or household breadwinners may be one of the biggest obstacles in containing the COVID-19 pandemic [15].

SARS-CoV-2 is composed of structural and nonstructural proteins. The structural proteins are the nucleocapsid protein (N), membrane protein (M), envelope protein (E), and spike protein (S), the latter being necessary for virus entry into the cell [7, 16]. Once the virus infects cells, an adequate immune response is important. The characteristic immune response that occurs in the host in response to COVID-19 is orchestrated by both adaptive and innate immune responses and is crucial for protection against SARS-CoV-2. Adaptative immunity is composed of cellular immunity and humoral immunity. In this regard, B lymphocytes, cells specialized in the production of antibodies to fight infection (IgM, IgG, IgA, IgD, and IgE) are the main effectors in the humoral response [17, 18]. However, although many antibodies against the pathogen can be generated during infection, not all of them can neutralize the virus. Only antibodies that block cell infiltration and viral replication are neutralizing antibodies (NAbs) [19].

Concerning the above, there are currently several specific serological tests for detecting NAbs, including the GenScript cPass assay, based on the enzyme-linked immunosorbent assay (ELISA) method. Its use was authorized in November 2020 by the U.S. Food and Drug Administration (FDA). This test evaluates the interaction between the receptor binding domain (RBD) and the angiotensin 2 receptor (ACE2). Therefore, this assay emulates the block of the entry of the virus into the cell (GenScript, Piscataway, NJ, U.S.A.) [20, 21].

One of the reasons why the pandemic has been perpetuated in Mexico is that the number of people infected with SARS-CoV-2 is underestimated, and there are no adequate strategies to detect asymptomatic cases [22]. It is, therefore, crucial to know the previous exposure to the virus through the seropositivity of anti-SARS-CoV-2 antibodies in young adults to assess whether there has been a previous exposure to the virus [23]. In addition, to date, some people have not been vaccinated or, despite being immunized, could present an infection that could even be asymptomatic [24]. For the above reasons, this study aimed to determine the presence of IgM, IgG, and NAbs against SARS-CoV-2 in unvaccinated young adults from northern Mexico who had or were unaware of having had a previous SARS-CoV-2 infection.

## 2. Materials and Methods

### 2.1. Capture of Subjects and Sample Collection

From July 2021 to August 2021, 171 subjects from the general population (unvaccinated young adults between 18 and 35 years old) residing in Navojoa, Sonora, Mexico, situated in the state of Sonora, north of Mexico, who had not received the SARS-CoV-2 vaccine at the time of enrolment were recruited for the study by using a random sample. Young adults of both sexes who were between the ages of 18 and 35 years old were eligible for this study. All participants completed an informed consent declaration after being included at the Universidad de Sonora, Unidad Regional Sur, Sonora State, Mexico. In addition, all subjects completed a structured survey to collect clinical and demographic information, a history of SARS-CoV-2 infection, and information on the symptoms of the illness for people who previously had COVID-19. From the above, we divided the participants into two groups as follows: (1) subjects unvaccinated who reported no history of SARS-CoV-2 infection, and (2) subjects unvaccinated who reported a prior COVID-19 infection. This classification was created based on the answers provided by individuals through a survey. In turn, based on the symptoms listed in the survey, subjects who had a previous SARS-CoV-2 infection were divided into four categories: asymptomatic, mild symptoms, moderate symptoms, and severe COVID-19.

Peripheral blood from all the subjects was obtained by venipuncture into vacutainer tubes without anticoagulant for serum collection, which was isolated by standard protocols, aliquoted, and stored at −80°C until use.

### 2.2. IgG/IgM against SARS-CoV-2 Antibodies Detection

Using a lateral flow immunoassay, the Certum IgG/IgM Rapid Test™ cassette (from the company All Test Biotech., Hangzhou, China 310018) kit, IgM, and IgG antibodies against SARS-CoV-2 were qualitatively determined. Both antibodies against spike (S) and nucleocapsid (N) were determined. This test uses a lateral flow immunoassay to distinguish between SARS-CoV-2 IgG (relative sensitivity >99.9%, relative specificity 98%) and IgM (relative sensitivity 85%, relative specificity 96%) antibodies. Positive was defined if the line stained for IgM, IgG (even though another antibody was negative), or both IgM/IgG for either N or S proteins. The manufacturer's recommendations for the protocol were followed.

### 2.3. Determination of NAbs

The manufacturer's recommendations for the ELISA cPass™ SARS-CoV-2 Neutralizing Antibody Detection Kit (GenScript, Piscataway, NJ, USA) were followed to determine the NAbs. It is a surrogate virus neutralization assay (sVNT). This assay determines the interaction between the RBD of the wild-type S protein and the ACE2 receptor, with the results reported as percentage inhibition. The assay cut-off value for the detection of SARS-CoV-2 neutralizing antibodies is 30% signal inhibition, indicating a positive result equal to or above 30% and a negative result below 30%. The manufacturer's recommendations for the protocol were followed. The percentage of signal inhibition is calculated using the following formula:

(1) $$\begin{array}{c} \%\;\text{signal}\;\text{inhibition}=\left(1-\frac{\text{OD}450\;\text{sample}}{\text{Average}\;\text{OD}450\;\text{negative}\;\text{control}}\right)\times 100\%. \end{array}$$

### 2.4. Statistical Analysis

GraphPad Prism v. 8.0 software was used for the statistical analysis, and a value of *p* < 0.05 was considered significant. Parameters with a normal distribution were expressed as means and standard deviations (S.D.). Non-normal distribution parameters are presented in medians and interquartile ranges (25–75). For the inferential statistics for two groups, the Student's *t*-test was performed, as was the ANOVA test for more than two groups (data with normal distribution). For nonparametric data, the comparison of two groups was performed by the Mann–Whitney *U* test, while more than two groups were compared by the Kruskal–Wallis. We used the nonparametric Wilcoxon signed-rank test to compare two groups of continuous values (data with no parametric distribution).

The concordance between the positivity of both antibody tests was evaluated using Cohen's kappa (*κ*) coefficient (McHugh, 2012). The interpretation was graded as follows: <0.20 no agreement, 0.21–0.40 least agreement, 0.41–0.60 weak agreement, 0.61–0.80 strong agreement, and 0.81–1.00 almost perfect agreement. Confidence intervals were calculated using the following formula:

(2) $$\begin{array}{c} \kappa –1.96\times \text{SE}\kappa \;\text{to}\;\kappa +1.96\times \text{SE}\kappa . \end{array}$$

## 3. Results

### 3.1. Clinical and Demographic Characteristics of the Subjects

A total of 171 young Mexican adults without prior vaccination and individuals of both genders (69% women and 31% men) with an age range of 18 to 35 years were included.  Table 1 shows the clinical and demographic data. Only 36 (21%) of the individuals included in the study self-reported a prior infection with SARS-CoV-2 while the remaining 135 (79%) subjects reported never having symptoms or having been diagnosed with COVID-19. Individuals were classified according to the clinical symptoms they presented during infection. Most individuals with a history of COVID-19 reported mild symptoms (80.5%): 13.8% of the subjects were asymptomatic, 80.5% had mild symptoms, and 2.8% reported moderate or severe symptoms (Table 1). No differences were found between the ages of the groups of young people with and without prior COVID-19, and no significant differences were observed between the genders of the two groups (Table 1).

### 3.2. Association between IgM/IgG Antibodies and Neutralizing Capacity of Unvaccinated Subjects

We observed that 42.6% of subjects were negative for the rapid IgM/IgG antibody test, and 57.3% of individuals were positive; of these, 87.8% were positive for IgG. In addition, of the total number of individuals included, it was found that 57.3% had NAbs and 42.7% were negative for NAbs. On the other hand, we compared the subjects who reported a previous COVID-19 infection and those who did not, and we observed that 86.1% and 49.6%, respectively, obtained a positive result in the rapid test, and we observed significant differences between both groups (*p* < 0.0001). When evaluating the levels of NAbs against SARS-COV-2, we observed that 91.7% of people who reported previous COVID-19 developed NAbs in response to infection. Of note, of the subjects without prior COVID-19, 49.6% had a positive rapid test result and 48.1% had NAbs (Table 2).

### 3.3. Comparison between the Results of the Certum IgG/IgM Rapid Test and the Percentage of NAbs against SARS-CoV-2

**Figure 1:**
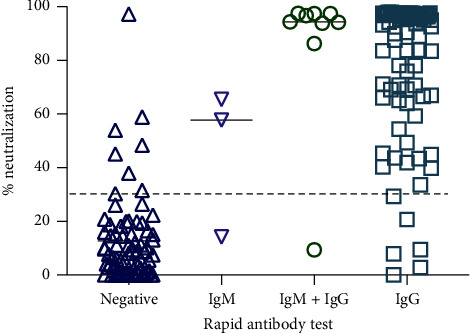
Percentage of neutralization and results of antibodies evaluated with the rapid test. The individuals were classified according to the results obtained in the rapid test.

To corroborate the results of the individuals who obtained different results between both tests (data in Table 2) and to evaluate the association between the IgG/IgM rapid test and NAbs, we performed a stratification according to the results obtained in the rapid test. Group 1: individuals with a negative result for the rapid test; group 2: IgM-positive individuals; group 3: IgM/IgG positive individuals; and group 4 was composed of IgG positive individuals. We found that most individuals (83.3%) who developed NAbs were IgG-positive on the rapid test (*n* = 80). In addition, 8 individuals (8.3%) were IgM/IgG positive in the rapid test and had NAbs. In comparison, 8 individuals (8.3%) were negative in the test with neutralizing capacity, although they were not detected by the rapid test (Figure 1).

### 3.4. Comparison between NAbs in Young Adults with and without Prior COVID-19 (by Self-Report)

**Figure 2:**
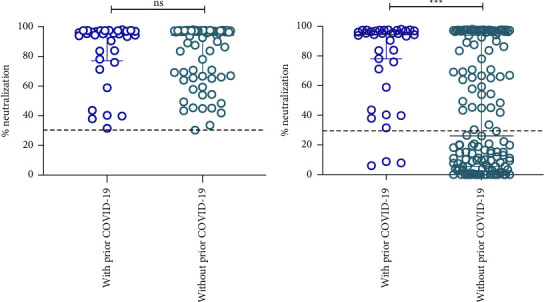
Comparison between the percentage of neutralization in young adults with and without prior COVID-19 (by self-report). Individuals were classified with and without COVID-19 based on their report in the survey. The *p* value was calculated using the Mann–Whitney *U* test. The data are provided as medians and interquartile ranges. ^*∗∗∗*^, *p* < 0.0001; Ns, no significance. (a) Comparison only between individuals with positive neutralizing antibodies. (b) Comparison between positive and negative neutralizing antibodies.

We compared the neutralizing capacity only in subjects positive for NAbs between individuals who reported a history of COVID-19 and those who reported no history of the disease. We observed that the neutralizing capacity in both study groups was very similar if subjects without NAbs (<30%) were excluded (Figure 2(a)). These results show that both groups had a previous infection and produced NAbs, even though one group did not show any symptoms.

On the other hand, after comparing only young adults positive for NAbs, we sought to compare the NAbs of individuals with and without prior COVID-19. We observed that in these two groups, the levels of NAbs were heterogeneous, and the analysis showed that there was a significant difference (*p* < 0.0001) between the groups (Figure 2(b)). The median percentage of neutralization in subjects who reported not having had COVID-19 before sampling (26.07%) was lower compared to the group of those who reported having had the disease (94.3%); however, almost half (48.1%) of the group without prior COVID-19 was positive for neutralizing antibodies.

### 3.5. Concordance between the Certum IgG/IgM Rapid Test Kit and cPass™ SARS-CoV-2 Neutralization Antibody Detection Kit

To evaluate the concordance between both tests, we stratified the individuals into two groups: 1: individuals positive to the rapid test (including positive IgM, IgG, and IgM/IgG); and group 2: individuals who developed NAbs. From this stratification, we performed Cohen's Kappa method, obtaining a Kappa value of 0.809, a standard error of 0.045, and a significance of 0.001 (Table 3). In addition, we observed that 65 individuals were without NAbs and negative for the rapid test, while only eight individuals who were negative for the rapid test had NAbs. Similarly, we obtained 90 individuals who had NAbs and were positive for the rapid test and only eight who were positive for the rapid test but did not have NAbs. This indicates that 90.6% (155/171) of individuals coincide with the results of both tests.

## 4. Discussion

It has been widely reported that droplets and aerosols easily transmit COVID-19 from person to person. Among the most common symptoms of COVID-19 are cough, fever, sore throat, headache, or severe symptoms that can lead to death [7]. Despite the above, it has also been reported that approximately ≈40.5% of SARS-CoV-2 infections can be asymptomatic [10].

In this study, we analyzed samples from unvaccinated young adults from Northern Mexico to determine the seroprevalence of anti-SARS-CoV-2 antibodies.

Antibodies against SARS-CoV-2 are highly heterogeneous in titers; higher antibody titers have been associated with disease severity, gender, and age. In the present study, we did not find significant differences when comparing male and female genders (data not shown), which differs from other studies where a difference was observed in the course of the disease and higher antibody titers in men [25, 26]. This difference could be explained by the fact that the median age of women was 41 years, while in our study, the median age of women was 25 years. The differences observed in these previous studies with older women could be mainly due to hormonal changes, such as decreased estrogen levels. These changes can influence the production of antibodies affecting B cell maturation, differentiation, activity, and survival, as estrogens can stimulate higher concentrations of antibodies in response to viral infections [27]. The fact that the women in this study are younger and are not undergoing these hormonal changes may explain why our results differ and no association with gender was found [28, 29].

To evaluate prior COVID-19 infections in young adults, primarily students and workers who maintain contact with many people, we conducted a structured survey through which 21.05% of individuals self-reported a previous COVID-19 infection, and 78.94% did not report a history of the disease. Surprisingly, when we analyzed the samples, we realized that many individuals without a history of COVID-19 (self-reported) were positive for anti-SARS-CoV-2 antibodies, and many of them also presented titles of NAbs. Those subjects indicated that they had never presented symptoms related to COVID-19; therefore, it can be assumed that they were asymptomatic cases.

Some factors increase the risk of virus exposure in asymptomatic infection, among them working outside the home; since the individual does not have symptoms, they do not stop working and continue with their daily life. Another important factor is domestic contact. A systematic review by Madewell et al. [30] showed increased transmissibility of emerging SARS-CoV-2 variants of concern in the confines of the home, and vaccination reduced these results. The above is important because asymptomatic cases have the potential to perpetuate infections among vulnerable individuals or those who might eventually develop symptomatic disease. Moreover, this is crucial considering studies suggesting that reinfections could elevate the long-term risk of COVID-19, albeit to a lesser extent in mild or asymptomatic infections [31].

The delta variant was initially identified in India in the early months of 2021 [32]. However, in Mexico, the Delta variant was first detected at the end of August 2021 [33]. Furthermore, Delta became the predominant variant in the latter months of 2021 [33]. Additionally, the first case of the Omicron variant in Mexico was identified on November 16, 2021 [34]. Consequently, our study period did not encompass the transmission periods of the Delta and Omicron variants. Nevertheless, it is imperative to highlight that these variants played a significant role in the elevated number of infections during the pandemic. Simultaneously, our results remain pertinent, as even outside the timeframe of these variants, a noteworthy incidence of asymptomatic infections was observed. This observation suggests the potential for an increase in case numbers with the emergence of new variants.

A study by Zou et al. detected a similar viral load between symptomatic and asymptomatic individuals, suggesting a high potential for transmission from individuals who did not present symptoms or were minimally symptomatic [35].

In this sense, our results suggest that young subjects who did not develop COVID-19 symptoms could be responsible for the silent spread of SARS-CoV-2 because they did not receive medical attention and had no home isolation. This is consistent with other findings in different populations, which raises the possibility that the spread of the virus asymptomatically may be greater than expected [36]. In addition, this study highlights the necessity of conducting random test screenings in busy areas or workplaces.

When we compared NAbs percentage in individual positive to anti-SARS-CoV-2 antibodies (with or without a COVID-19 history, by self-report), we did not observe significant differences; therefore, this suggests that NAbs are synthesized even in an asymptomatic individual. Previous studies support the above, showing that NAb titers are maintained independently of symptoms [37].

For the above reasons, of the individuals who had NAbs, we stratified individuals with symptoms and those without symptoms. Of 98 individuals with neutralizing capacity, 33 presented symptoms (87.9% with mild symptoms, 2.8 with moderate, and 2.8 with severe symptoms), and 65 did not report any clinical signs. However, when we compared asymptomatic individuals with symptomatic individuals, we did not find a significant difference in the percentage of neutralization between these two groups. This confirms that both groups generated neutralizing antibodies even without being aware that they had suffered from the disease. This is consistent with other studies where it was observed that young adults with an asymptomatic SARS-CoV-2 infection had memory B cell responses similar to those seen with a moderately symptomatic infection [38].

Of the individuals with NAbs, we observed that the majority were also IgG positive; however, we observed that eight individuals negative to the rapid test were positive for NAbs; it could be due to the fact that the cPass test is not specific to any isotype (i.e., isotype agnostic), so it could be IgA antibodies, which the rapid test does not detect [20]. We observed that 7 of these 8 individuals negative to a rapid test but positive to NAbs have a low neutralizing capacity (30–<60% of neutralizing antibodies). We suggest that these could be of the IgA isotype; this could be supported by a study that demonstrates a significant response of antibodies with a neutralizing capacity of the IgA isotype but that decreased faster than the NAbs of the IgG type [39]. In this regard, we also found that 98 out of 171 individuals had NAbs; of these, 18 individuals had low NAbs titers (30–<60%), which could be because the titers of these antibodies decreased over time [40, 41]. Furthermore, it has been shown that individuals with mild or asymptomatic SARS-CoV-2 infections had lower antibody titers and SARS-CoV-2 NAbs as well as greater reinfection rates [42].

On the other hand, we also observed positive IgM/IgG and IgG individuals without NAbs; this could be the case of individuals who develop antibodies but do not prevent viral replication and therefore do not confer long-term immunity.

Despite the decrease in COVID-19 cases, it is difficult to rule out a possible increase in infections; therefore, it is important to continue monitoring antibodies to determine seroprevalence in vaccinated or unvaccinated people and define an accessible and reliable test, as well as the protection provided by antibodies acquired through vaccination to contribute to the resolution of the pandemic. Consequently, it could help to prevent and control infections not yet identified. For this reason, we decided to compare the findings of the two tests utilized in the study, and we found some similarities. We obtained an agreement of 90.6%, where 155 individuals agreed on the results of both tests; therefore, we evaluated the concordance between both tests. According to Cohen's Kappa interpretation, a value ≤ 0 indicates no agreement, 0.01–0.20 indicates none to a slight agreement, 0.21–0.39 is a minimal agreement, 0.40– 0.59 is a weak agreement, 0.60–0.79 is a moderate agreement, 0.80–0.90 is a strong agreement, and above 0.90 is almost perfect agreement [43]. In this sense, our results indicate a strong concordance between seropositivity to the rapid test and the neutralizing antibody test. Furthermore, in many countries, including Mexico, PCR testing to determine SARS-CoV-2 infection status has focused mainly on symptomatic patients. Therefore, this result is significant because, in Mexico, rapid tests for the detection of antibodies against SARS-CoV-2 are more accessible than neutralization tests and offer results in less than 30 minutes. Currently, a large part of the population has already been exposed to the virus or has been vaccinated, which may lead to the conclusion that most of these people have developed detectable antibodies at some point in time against some variant of SARS-CoV-2. Despite the above, there is evidence of a decrease in antibodies over time [44, 45], so it is important to highlight that screening tests continue to be a plausible strategy to detect asymptomatic cases or recent infections; therefore, the results obtained in this study on the concordance between the two tests are still widely relevant. It is important to consider that the use of a validated and certified rapid test alone or combined with molecular tests is an effective and accessible strategy to obtain a broader image of the prevalence of antibodies in people with previous exposure to the virus or with a disease in progress, respectively, and they could be useful for public health surveillance.

A limitation of this study is the lack of a timely diagnosis for individuals who had antibodies against SARS-CoV-2, which would allow us to observe a titer change over time. In addition, the overall sample size, especially the number of individuals with moderate and severe symptoms, was too few to compare the NAbs between the different groups of symptomatic subjects. Studies with a larger sample size are needed to corroborate these findings and more accurately estimate the percentage of asymptomatic individuals in this age group.

In later works, it will be essential to evaluate the titers of antibodies generated by vaccination and determine specific antibodies against the N protein in vaccinated individuals to differentiate them from a natural infection. In addition, it is also relevant to pay attention to the cellular immune response and its connection to the humoral immune response. Due to the fact that we observed eight individuals negative for the rapid test but positive for NAbs, it would be important to determine the subtypes of antibodies against the virus. The quantification of proinflammatory cytokines and their correlation with the severity of the disease and the anti-SARS-CoV-2 antibody titers will provide interesting results that will help us understand the disease's pathogenesis and the protection that could be given after an infection, whether symptoms are present or not.

## 5. Conclusions

Most young people who reported not having a prior infection were seropositive for anti-SARS-CoV-2 antibodies, indicating a very high number of asymptomatic for COVID-19 in this group. This is important because this group can be a silent spread of the SARS-CoV-2 in Mexico and could be partially contributing to maintaining new COVID-19 cases.

## Figures and Tables

**Table 1:** Clinical and demographic characteristics of the study groups.

Parameter	Total unvaccinated young adults *n* = 171	Prior COVID-19 by self-report		*p* value
		Yes 21.05% (*n* = 36)	No 78.94% (*n* = 135)	
Demographic				
Age (years)^a^	25 (18–35)	25 (18–35)	24 (18–35)	0.704
Gender^b^				
Female	69 (118/171)	75 (27/36)	67.4 (91/135)	0.381
Male	31 (53/171)	25 (9/36)	32.6 (44/135)	
Clinical symptoms^b^				
Asymptomatic	—	13.8 (7/36)	—	—
Mild	—	80.5 (29/36)	—	
Moderate	—	2.8 (1/36)	—	
Serious	—	2.8 (1/36)	—	

^a^Median; ^b^percentage data. *p* values were calculated by the Mann–Whitney *U* and chi-square test (median (IQR)).

**Table 2:** Rapid test and NAbs for SARS-CoV-2.

Test	Total unvaccinated young adults *n* = 171	Prior COVID-19 by self-report		*p* value
		Yes 21.05% (*n* = 36)	No 78.94% (*n* = 135)	
Rapid antibody test				
Negative	42.6% (73/171)	13.9% (5/36)	50.4% (68/135)	<0.0001
Positive	57.3% (98/171)	86.1% (31/36)	49.6% (67/135)	
IgM	3.1% (3/98)	0% (0/31)	4.5% (3/67)	0.488
IgM + IgG	9.2% (9/98)	9.7% (3/31)	8.9% (6/67)	
IgG	87.8% (86/98)	90.3% (28/31)	86.6% (58/67)	
Neutralizing antibodies				
Negative (<30%)	42.7% (73/171)	8.3% (3/36)	51.9% (70/135)	<0.0001
Positive (>30%)	57.3% (98/171)	91.7% (33/36)	48.1% (65/135)	
% neutralization	48.36 (9.415–96.01)	94.3 (68.14–96.96)	26.07 (6.035–93.130)	<0.0001

*p*-values were calculated by Fisher's exact, the Mann–Whitney *U* (%(*n*)) or chi-square test for the groups (median (IQR)).

**Table 3:** Kappa value for individuals positive to the rapid test and NAbs.

Kappa	SE	CI (95.0%)		*p* value
0.809	0.045	0.7208	0.8972	0.001

SE, standard error; CI, confidence interval.

## Data Availability

The data from this study are available from the corresponding author upon reasonable request.

## References

[1] Chen Y., Zhao X., Zhou H., Zhu H., Jiang S., Wang P. (2022). Broadly neutralizing antibodies to SARS-CoV-2 and other human coronaviruses. *Nature Reviews Immunology*.

[2] Lu R., Zhao X., Li J. (2020). Genomic characterisation and epidemiology of 2019 novel coronavirus: implications for virus origins and receptor binding. *The Lancet*.

[3] Gorbalenya A. E., Baker S. C., Baric R. S. (2020). The species severe acute respiratory syndrome-related coronavirus: classifying 2019-nCoV and naming it SARS-CoV-2. *Nature Microbiology*.

[4] Cucinotta D., Vanelli M. (2020). WHO declares COVID-19 a pandemic. *Acta Biomedica Atenei Parmensis*.

[5] Ortiz-Prado E., Simbaña-Rivera K., Gómez- Barreno L. (2020). Clinical, molecular, and epidemiological characterization of the SARS-CoV-2 virus and the coronavirus disease 2019 (COVID-19), a comprehensive literature review. *Diagnostic Microbiology and Infectious Disease*.

[6] Gandhi R. T., Lynch J. B., del Rio C. (2020). Mild or moderate covid-19. *New England Journal of Medicine*.

[7] Harrison A. G., Lin T., Wang P. (2020). Mechanisms of SARS-CoV-2 transmission and pathogenesis. *Trends in Immunology*.

[8] Fernandes T. J., Ogrzewalska M., Martins E. B., De Siqueira M. A. M. T., Brasil P., Calvet G. A. (2023). Oral manifestations of COVID-19 in unvaccinated patients: a cross-sectional study. *BMC Oral Health*.

[9] Wang B., Andraweera P., Elliott S. (2023). Asymptomatic SARS-CoV-2 infection by age: a global systematic review and meta-analysis. *The Pediatric Infectious Disease Journal*.

[10] Ma Q., Liu J., Liu Q. (2021). Global percentage of asymptomatic SARS-CoV-2 infections among the tested population and individuals with confirmed COVID-19 diagnosis: a systematic review and meta-analysis. *Journal of the American Medical Association Network Open*.

[11] Yanes-Lane M., Winters N., Fregonese F. (2020). Proportion of asymptomatic infection among COVID-19 positive persons and their transmission potential: a systematic review and meta-analysis. *PLoS One*.

[12] Álvarez-del Río B., Sánchez-de Prada L., Álvaro-Meca A. (2023). Prevalence and cost of hospitalized patients with asymptomatic COVID-19 in 2020 in Spain. *Frontiers in Public Health*.

[13] Ochoa-Hein E., Rajme-López S., Leal-Morán P. E. (2021). Factors associated with COVID-19 and asymptomatic carriage in healthcare workers of a COVID-19 hospital. *Journal of clinical research; organ of the Hospital for Nutritional Diseases*.

[14] Johansson M. A., Quandelacy T. M., Kada S. (2021). SARS-CoV-2 transmission from people without COVID-19 symptoms. *Journal of the American Medical Association Network Open*.

[15] Das A. K., Chandra K., Dudeja M., Aalam M. K. (2022). Asymptomatic SARS-COV-2 carriage and sero-positivity in high risk contacts of COVID-19 cases. *Indian Journal of Medical Microbiology*.

[16] Walls A. C., Park Y.-J., Tortorici M. A., Wall A., McGuire A. T., Veesler D. (2020). Structure, function, and antigenicity of the SARS-CoV-2 spike glycoprotein. *Cell*.

[17] Chvatal-Medina M., Mendez-Cortina Y., Patiño P. J., Velilla P. A., Rugeles M. T. (2021). Antibody responses in COVID-19: a review. *Frontiers in Immunology*.

[18] Ou X., Liu Y., Lei X. (2020). Characterization of spike glycoprotein of SARS-CoV-2 on virus entry and its immune cross-reactivity with SARS-CoV. *Nature Communications*.

[19] Garcia-Beltran W. F., Lam E. C., Astudillo M. G. (2021). COVID-19-Neutralizing antibodies predict disease severity and survival. *Cell*.

[20] Taylor S. C., Hurst B., Charlton C. L. (2021). A new SARS-CoV-2 dual-purpose serology test: highly accurate infection tracing and neutralizing antibody response detection. *Journal of Clinical Microbiology*.

[21] Papenburg J., Cheng M. P., Corsini R. (2021). Evaluation of a commercial culture-free neutralization antibody detection kit for severe acute respiratory syndrome-related coronavirus-2 and comparison with an antireceptor-binding domain enzyme-linked immunosorbent assay. *Open Forum Infectious Diseases*.

[22] Ibarra-Nava I., Cardenas-de la Garza J. A., Ruiz-Lozano R. E., Salazar-Montalvo R. G. (2020). Mexico and the COVID-19 response. *Disaster Medicine and Public Health Preparedness*.

[23] Sah P., Fitzpatrick M. C., Zimmer C. F. (2021). Asymptomatic SARS-CoV-2 infection: a systematic review and meta-analysis. *Proceedings of the National Academy of Sciences*.

[24] North C. M., Barczak A., Goldstein R. H. (2022). Determining the incidence of asymptomatic SARS-CoV-2 among early recipients of COVID-19 vaccines (DISCOVER-COVID-19): a prospective cohort study of healthcare workers before, during and after vaccination. *Clinical Infectious Diseases*.

[25] Markmann A. J., Giallourou N., Bhowmik D. R. (2021). Sex disparities and neutralizing-antibody durability to SARS-CoV-2 infection in convalescent individuals. *mSphere*.

[26] Mukherjee S., Pahan K. (2021). Is COVID-19 gender-sensitive?. *Journal of Neuroimmune Pharmacology*.

[27] Pinna G. (2021). Sex and COVID-19: a protective role for reproductive steroids. *Trends in Endocrinology and Metabolism*.

[28] Harding A. T., Heaton N. S. (2022). The impact of estrogens and their receptors on immunity and inflammation during infection. *Cancers*.

[29] Wend K., Wend P., Krum S. A. (2012). Tissue-specific effects of loss of estrogen during menopause and aging. *Frontiers in Endocrinology*.

[30] Madewell Z. J., Yang Y., Longini I. M., Halloran M. E., Dean N. E. (2022). Household secondary attack rates of SARS-CoV-2 by variant and vaccination status: an updated systematic review and meta-analysis. *Journal of the American Medical Association Network Open*.

[31] Boufidou F., Medić S., Lampropoulou V., Siafakas N., Tsakris A., Anastassopoulou C. (2023). SARS-CoV-2 reinfections and long COVID in the post-omicron phase of the pandemic. *International Journal of Mathematics and Statistics*.

[32] Earnest R., Uddin R., Matluk N. (2022). Comparative transmissibility of SARS-CoV-2 variants Delta and alpha in new england, USA. *Cell Reports Medicine*.

[33] Aguayo-Acosta A., Oyervides-Muñoz M. A., Rodriguez-Aguillón K. O. (2024). Omicron and Delta variant prevalence detection and identification during the fourth COVID-19 wave in Mexico using wastewater-based epidemiology. *IJID Regions*.

[34] Cedro-Tanda A., Gómez-Romero L., De Anda-Jauregui G. (2022). Early genomic, epidemiological, and clinical description of the SARS-CoV-2 Omicron variant in Mexico city. *Viruses*.

[35] Zou L., Ruan F., Huang M. (2020). SARS-CoV-2 viral load in upper respiratory specimens of infected patients. *New England Journal of Medicine*.

[36] Almadhi M. A., Abdulrahman A., Sharaf S. A. (2021). The high prevalence of asymptomatic SARS-CoV-2 infection reveals the silent spread of COVID-19. *International Journal of Infectious Diseases*.

[37] Reynolds C. J., Swadling L., Gibbons J. M. (2020). Discordant neutralizing antibody and T cell responses in asymptomatic and mild SARS-CoV-2 infection. *Science Immunology*.

[38] Kato Y., Bloom N. I., Sun P. (2022). Memory B-cell development after asymptomatic or mild symptomatic SARS-CoV-2 infection. *The Journal of Infectious Diseases*.

[39] Takamatsu Y., Omata K., Shimizu Y. (2022). SARS-CoV-2-Neutralizing humoral IgA response occurs earlier but modest and diminishes faster compared to IgG response. *Microbiology Spectrum*.

[40] Ibarrondo F. J., Fulcher J. A., Goodman-Meza D. (2020). Rapid decay of anti–SARS-CoV-2 antibodies in persons with mild covid-19. *New England Journal of Medicine*.

[41] Long Q.-X., Tang X.-J., Shi Q.-L. (2020). Clinical and immunological assessment of asymptomatic SARS-CoV-2 infections. *Nature Medicine*.

[42] Lazor-Blanchet C., Zygoura P., Dafni U. (2022). Low neutralizing antibody titers after asymptomatic or non-severe SARS-CoV-2 infection over a 6-month assessment period. *Journal of Infection*.

[43] McHugh M. L. (2012). Interrater reliability: the kappa statistic. *Biochemical Medicine*.

[44] Röltgen K., Powell A. E., Wirz O. F. (2020). Defining the features and duration of antibody responses to SARS-CoV-2 infection associated with disease severity and outcome. *Science Immunology*.

[45] Lyke K. E., Atmar R. L., Islas C. D. (2022). Rapid decline in vaccine-boosted neutralizing antibodies against SARS-CoV-2 Omicron variant. *Cell Reports Medicine*.

